# The impact of cardiac rehabilitation on rehospitalization and mortality rates in heart failure with preserved ejection fraction

**DOI:** 10.3389/fcvm.2026.1799035

**Published:** 2026-05-28

**Authors:** Fenghong Liu, Qiyao Wang, Shanshan Han, Manman Zhang, Xiaoyu Bai, Cuihua Wang

**Affiliations:** 1 Department of Cardiac Functional Rehabilitation, The First Hospital of Hebei Medical University, Shijiazhuang City, China; 2 Arrhythmia Center, The First Hospital of Hebei Medical University, Shijiazhuang City, China

**Keywords:** cardiac rehabilitation, exercise, heart failure with preserved ejection fraction, mortality, rehospitalization rate

## Abstract

**Introduction:**

Heart failure with preserved ejection fraction (HFpEF) is associated with substantial functional limitation and recurrent hospitalization, while effective pharmacological options remain limited. Exercise-based cardiac rehabilitation (CR) has been proposed as a non-pharmacological strategy; however, its effects on clinical and functional outcomes in HFpEF remain incompletely defined.This meta-analysis aimed to systematically assess the impact of exercise-based CR on rehospitalization, mortality, functional capacity, quality of life, and cardiac function in patients with HFpEF.

**Methods:**

A systematic review was conducted according to PRISMA guidelines, with electronic database searches performed through December 2024.Nineteen randomized controlled trials involving 2,025 participants were included. Meta-analyses were performed using RevMan 5.4, with effect estimates expressed as risk ratios (RR) or mean differences (MD) with 95% confidence intervals (CI).

**Results:**

Exercise-based CR did not significantly reduce all-cause mortality (RR 1.13, 95% CI 0.77–1.66), but was associated with a significantly lower risk of rehospitalization (RR 0.52, 95% CI 0.38–0.72). Significant improvements were observed in functional exercise capacity, including 6-minute walk distance (MD 36.89m) and peak oxygen uptake (VO₂peak; MD 2.14mL/kg/min), as well as physical functioning assessed by the SF-36. Exercise-based CR was also associated with a modest reduction in left atrial volume index, whereas left ventricular ejection fraction remained unchanged. In addition, peak heart rate significantly improved (MD 7.74 bpm, 95% CI 3.68–11.80). Data on blood pressure, lipid profile, glycemic control, and cardiovascular mortality were limited or inconsistently reported, precluding meta-analysis.

**Conclusion:**

Exercise-based cardiac rehabilitation improves functional capacity,cardiorespiratory fitness,and physical quality of life in patients with HFpEF and reduces rehospitalization risk, although no mortality benefit was observed.

**Systematic Review Registration:**

https://www.crd.york.ac.uk/prospero/display_record.php?RecordID=1069784, PROSPERO CRD420251069784.

## 1 Introduction

Heart failure with preserved ejection fraction (HFpEF) is a complex and heterogeneous clinical syndrome defined by the presence of typical symptoms and signs of heart failure in patients with a normal or near-normal left ventricular ejection fraction (1). Unlike heart failure with reduced ejection fraction(HFrEF), HFpEF is characterized predominantly by abnormalities in diastolic function, including impaired myocardial relaxation, increased ventricular stiffness, and abnormal ventricular–arterial coupling. These pathophysiological alterations lead to elevated left ventricular filling pressures, particularly during exertion, and contribute directly to exercise intolerance and dyspnea, which are hallmarks of the disease (2).

Over the past several decades, HFpEF has emerged as the most common form of heart failure, especially among older adults and individuals with cardiometabolic comorbidities such as hypertension, obesity, diabetes mellitus, and atrial fibrillation (3). Epidemiological studies indicate that HFpEF now accounts for approximately half of all heart failure cases and that its prevalence continues to rise as populations age. Importantly, the prognosis of HFpEF remains poor, with rates of hospitalization, recurrent decompensation, and mortality that are comparable to those observed in HFrEF. In addition to adverse clinical outcomes, HFpEF is associated with marked impairments in quality of life and substantial socioeconomic burden (4, 5). In addition to adverse clinical outcomes, HFpEF is associated with marked impairments in quality of life and substantial socioeconomic burden.

Therapeutic options for HFpEF are limited. Although guideline-directed medical therapy emphasizes symptom relief, blood pressure control, and management of comorbid conditions, its effect on long-term outcomes has been modest. While recent trials have suggested potential benefits of sodium–glucose cotransporter 2 inhibitors in HFpEF, there remains a clear need for complementary treatment strategies that address the functional limitations and recurrent hospitalizations experienced by this patient population (6). Consequently, non-pharmacological interventions have attracted increasing attention.

Cardiac rehabilitation (CR) is a comprehensive non-pharmacological intervention that integrates structured exercise training, medical supervision, and lifestyle modification, typically delivered by a multidisciplinary team. Exercise training constitutes the core component of CR and is designed to improve physical capacity, optimize cardiovascular risk factors, and promote long-term behavioral change (7). In HFpEF, where peripheral limitations substantially contribute to exercise intolerance, CR has gained increasing attention (8, 9). Evidence from randomized controlled trials and meta-analyses indicates that exercise training significantly improves functional capacity in patients with HFpEF. Consistent benefits have been observed in peak oxygen uptake (VO₂peak) and 6 min walk distance (6MWD), reflecting improved cardiorespiratory fitness and submaximal exercise performance (8). Exercise interventions have also been associated with improvements in quality of life and physical functioning, potentially mediated through enhanced endothelial function, reduced systemic inflammation, and improved skeletal muscle and myocardial metabolism (9).

However, despite these functional benefits, the impact of exercise-based cardiac rehabilitation on clinical outcomes such as rehospitalization and mortality in HFpEF remains uncertain. Existing studies have produced inconsistent findings, often limited by small sample sizes and short follow-up durations. A previous meta-analysis by Pandey et al. (10) reported improvements in exercise capacity and quality of life but emphasized the need for further evidence regarding clinical endpointss (10).

Accordingly, this meta-analysis aimed to systematically evaluate the effects of exercise training–based cardiac rehabilitation on rehospitalization, all-cause mortality, exercise capacity, quality of life, and cardiac function in patients with HFpEF.

## 2 Methods

This systematic review and meta-analysis were conducted in accordance with the PRISMA statement for systematic reviews and meta-analyses. The procedure for this assessment has been recorded with PROSPERO (CRD420251069784).

### 2.1 Retrieval strategies and data sources

We systematically searched databases from their inception until December 31, 2024, including PubMed, Embase, Web of Science, Cochrane Library, China Knowledge Network, Wei pu Data, Wan fang Data, and China Biomedical Database. We utilized keywords and subject terms such as “heart failure with preserved ejection fraction,” “heart failure with normal ejection fraction,” “diastolic heart failure,” “HFpEF,” “exercise,” “training,” “rehabilitation therapy,” and “randomized controlled trial.” Additionally, we manually searched relevant reviews and the reference lists of included studies to identify available papers and ensure that no literature was overlooked.

### 2.2 Research selection (inclusion and exclusion criteria)

The inclusion criteria for this literature review are as follows: (1) The research subjects are patients with a clearly diagnosed HFPEF. (2) The intervention consists of cardiac rehabilitation, which includes a comprehensive exercise training program incorporating aerobic exercise, resistance training, and other modalities. It may be complemented by education, risk factor management, lifestyle counseling, and regular follow-ups. (3) There is a control group that receives either routine treatment or a placebo intervention. (4) The research design is a randomized controlled trial (RCT). (5) The study reports at least one index related to evaluating the effects of exercise training, such as rehospitalization rates, mortality, exercise tolerance, quality of life, or diastolic function. (6) The research includes a follow-up period of a specified duration. The exclusion criteria for this literature are: (1) heart failure patients with reduced ejection fraction or those with unreported LVEF; (2) studies with design flaws, such as non-randomized controlled trials, retrospective studies, or those lacking control groups; (3) unclear intervention measures or studies that include non-exercise interventions; (4) incomplete data or studies that do not allow for data extraction, making quantitative analysis impossible; (5) animal experiments, review articles, and non-English publications.

### 2.3 Data extraction

The extracted data will include basic information about the study, such as the first author, publication year, sample size, and patient characteristics; detailed information regarding the intervention measures, including the type and duration of exercise training; and the main outcome indicators of the study, such as rehospitalization rates, mortality rates, results from exercise tolerance tests, quality of life scores, and indicators of diastolic function. Additionally, baseline medication use was recorded, including beta-blockers, angiotensin-converting enzyme inhibitors/angiotensin receptor blockers (ACEIs/ARBs), diuretics, statins, calcium channel blockers, and other relevant medications. Cardiometabolic outcomes, including blood pressure, lipid profiles, and glycemic control, were not pooled due to substantial methodological heterogeneity and inconsistent reporting across studies. In particular, blood pressure measurements varied between resting and exercise (peak) conditions, limiting comparability. Therefore, these outcomes were qualitatively assessed but not included in the quantitative synthesis.

### 2.4 Quality assessment

The methodological quality of included studies was independently assessed by two reviewers using the Cochrane Risk of Bias assessment tool. The following domains were evaluated: random sequence generation, allocation concealment, blinding of participants and personnel, blinding of outcome assessment, completeness of outcome data, selective outcome reporting, and other potential sources of bias. Each domain was rated as low, unclear, or high risk of bias. Any disagreements were resolved through consensus.

### 2.5 Statistical analysis

Statistical analyses were conducted using Review Manager software (RevMan version 5.4). For continuous outcomes, pooled effects were expressed as mean differences (MD) with corresponding 95% confidence intervals (CI). For dichotomous outcomes, effect estimates were reported as risk ratios (RR) with 95% CI. When available, changes from baseline to follow-up were preferentially used in the analysis. Between-study heterogeneity was assessed using the I^2^ statistic. An I^2^ value below 50% was considered indicative of low heterogeneity, and a fixed-effect model was applied in such cases. When heterogeneity was moderate or substantial (I^2^ ≥ 50%), a random-effects model was used.

Pre-specified subgroup analyses were performed to explore potential sources of heterogeneity. These analyses were stratified according to outcome type, duration of follow-up (≤6 months vs. >6 months), and intervention characteristics, distinguishing between exercise-only programs and comprehensive cardiac rehabilitation interventions that incorporated education or multidisciplinary support. Additional subgroup analyses examined the potential influence of baseline medication use, including beta-blockers, ACEIs/ARBs, and diuretics, with detailed results provided in Supplementary File S1. Sensitivity analyses were conducted by sequentially excluding individual studies to evaluate the robustness of the pooled estimates and identify studies with disproportionate influence on heterogeneity. Subgroup analyses based on patient-level characteristics (e.g., age, diabetes status, and other clinical characteristics) were not performed due to the lack of stratified outcome data and absence of reported interaction analyses in the included trials.

## 3 Outcomes

### 3.1 Included studies

**Figure 1:**
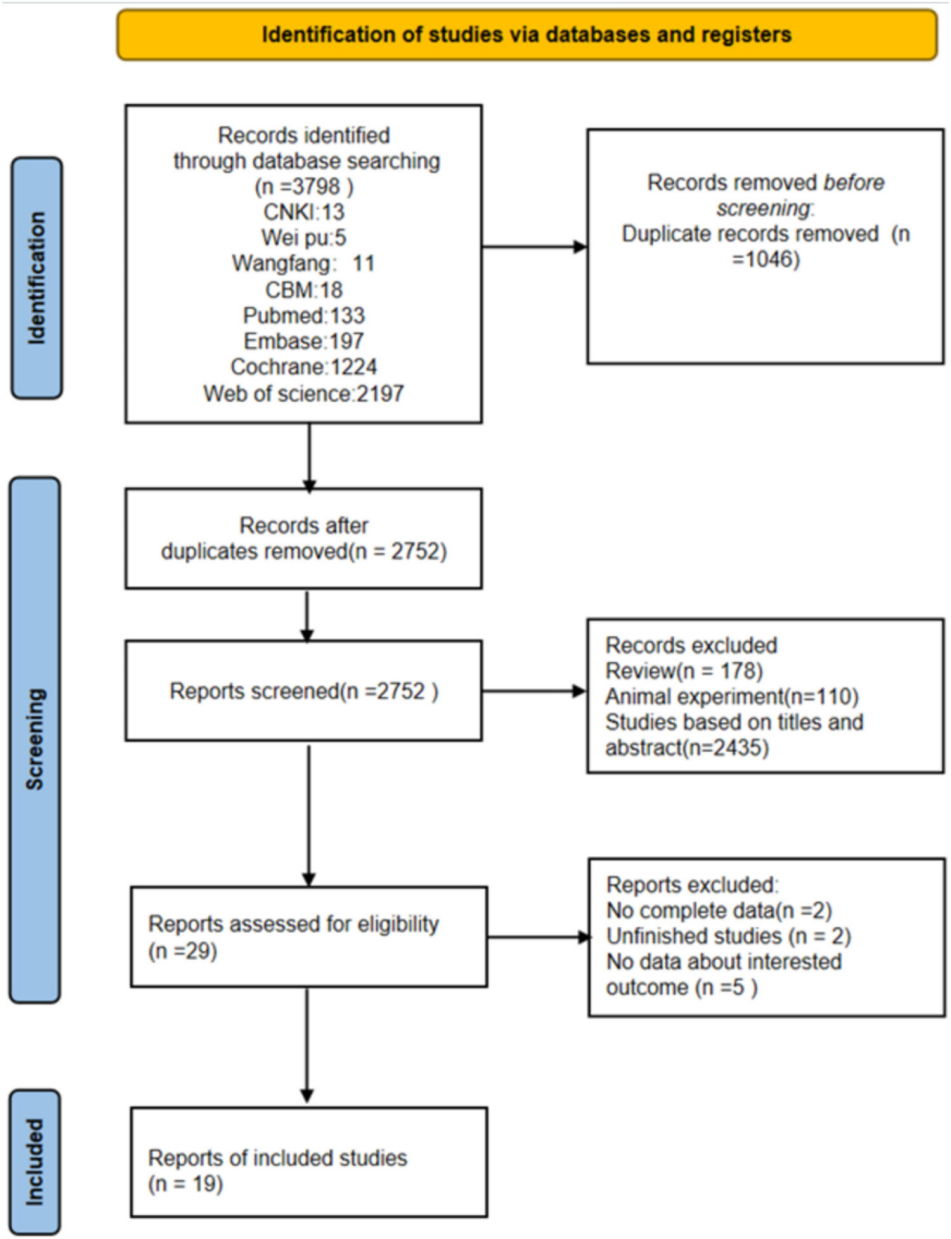
Flowchart of the PRISMA study selection process.

Through a comprehensive search of databases and manual retrieval, a total of 3,798 articles were identified. After eliminating duplicates, 2,752 articles remained. A subsequent screening of titles and abstracts led to the exclusion of 2,723 documents that did not meet the inclusion criteria. We then reviewed the full texts of the remaining articles, further excluding 10. Ultimately, 19 documents were determined to meet the inclusion criteria. The flowchart summarizing the research selection process is presented in Figure 1.

### 3.2 Characteristics of included literature

Nineteen randomized controlled trials were included in this review (11–29), encompassing a total of 2,025 subjects. Baseline characteristics revealed that the average age of patients ranged from 61.8 ± 2.9 years to 76.9 ± 5.67 years, with the majority of patients aged between 60 and 70 years. LVEF was predominantly above 45%.

The primary intervention across the included trials was CR, with exercise training serving as the central therapeutic component. Most studies focused on supervised exercise programs, typically involving structured aerobic training alone or in combination with resistance exercise. In contrast, several trials—such as those conducted by Andryukhin et al. (12), Alonso et al. (11), and Antonicelli et al. (13)—adopted a more comprehensive, multidisciplinary CR approach that integrated exercise training with patient education and cardiovascular risk factor management. Although the study by Kitzman et al. (22) incorporated certain multidisciplinary elements, exercise training remained the dominant intervention component. Participants in the control groups generally received standard medical or nursing care without prescribed exercise interventions. Follow-up durations across studies ranged from 3 to 18 months. Detailed baseline clinical characteristics are presented in Table 1, while Table 2 summarizes the specific exercise protocols, intervention durations, control group care, and reported outcomes.

**Table 1:** Basic characteristics of included studies.

study	Baseline sample size (intervention group/control group)	Follow-up sample size (intervention group/control group)	Age (years)	Gender (male/female)	LVEF(%)
Alonso et al. (11)	59 (25/34)	59 (25/34)	64.6 ± 9.3	32/27	55 ± 6
Andryukhin et al. (12)	85 (44/41)	85 (44/41)	67.2 ± 5.9	26/59	≥45%
Antonicelli et al. (13)	34 (170/173)	313 (150/163)	76.9 ± 5.67	195/148	48.4 ± 13.4
Baldassarri et al. (14)	62 (43/19)	62 (43/19)	64.6 ± 7.2	28/34	67.3 ± 6.8
Brubaker et al. (14)	116 (58/58)	99 (48/51)	69.75 ± 6.45	22/94	60.0 ± 0.1
Corsi et al. (16)	34 (175/174)	349 (149/155)	72.6 ± 8.1	166/183	≥45%
Edelmann et al. (17)	60 (41/19)	60 (41/19)	65 ± 7	24/36	67 ± 7
Fu et al. (18)	60 (30/30)	59 (30/29)	61.8 ± 2.9	38/22	57.6 ± 1.9
Haykowsky et al. (19)	40 (22/18)	40 (22/18)	69.1 ± 5.6	5/35	≥45%
Kitzman et al. (21)	53 (26/27)	46 (24/22)	69.5 ± 5.5	13/40	≥50%
Kitzman et al. (20)	63 (32/31)	The number of people for each indicator varies	70 ± 7	15/48	≥50%
Kitzman et al. (22)	349 (175/ 174)	The number of people for each indicator varies	72.7 ± 8.1	166/183	≥45%
Lang et al. (23)	50 (25/25)	48 (22/23)	73.9 ± 8.6	23/27	≥45%
Maldonado-Martín et al. (24)	47 (23/24)	47 (23/24)	Not mentioned	6/41	≥50%
Mueller et al. (25)	176 (58/60)	The number of people for each indicator varies	69.7 ± 8.2	59/117	≥50%
Palau et al. (27)	26 (14/12)	26 (14/12)	70.8 ± 9.3	13/13	Not mentioned
Palau et al. (26)	28 (15/13)	26 (13/13)	75 ± 9.3	11/17	67 ± 10
Smart et al. (28)	25 (12/13)	25 (12/13)	64.3 ± 6.8	13/25	57 ± 10
Sowa et al. (29)	34 (24/10)	34 (24/10)	71.2 ± 2.5	9/25	63.6 ± 2.6

**Table 2:** Interventions included in the study.

Study	Exercise Training Group Intervention	Intervention Type	Control Group Intervention	Follow-u*p* Duration	Outcomes
Alonso et al. (11)	HEART Camp multi- component intervention	Comprehensive CR	Enhanced routine care	6, 12, 18 months	6MWT
Andryukhin et al. (12)	4-week lifestyle & risk factor education, 6-month home exercise, 15–30 min/week consultation	Comprehensive CR	Russian national guidelines routine care	6,18 months	Rehospitalizations, deaths, 6MWT
Antonicelli et al. (13)	3-month hospital-supervised training (warm-up, exercise, cool-down), 3-month home remote-monitored training	Comprehensive CR	12-week routine medical management	3,6 months	Rehospitalizations, deaths, 6MWT
Baldassarri et al. (14)	1–4 weeks: increased aerobic endurance training; 5+ weeks: increased frequency, intensity & added resistance training	Exercise Only	Regular daily activities	3 months	6MWT, PeakVO2, LVAI
Brubaker et al. (15)	3-times/week supervised aerobic & resistance training	Exercise Only	Maintain activity, bi-weekly calls	4 months	6MWT, SF-36 Physical Function Scale, PeakVO2, VAT
Corsi et al. (16)	12-week, 3-days/week outpatient training for strength, balance, mobility, endurance	Comprehensive CR	Usual care	3,6 months	Deaths, 6MWT
Edelmann et al. (17)	32-rep structured exercise: 1–4 weeks aerobic endurance, 5 + weeks added resistance	Exercise Only	Daily activities & usual HFpEF care	3 months	6MWT, SF-36 Physical Function Scale, PeakVO2, LVEF, LVAI
Fu et al. (18)	3-times/week bicycle ergometer aerobic interval training	Exercise Only	General home health care	3 months	SF-36 Physical Function Scale, LVEF
Haykowsky et al. (19)	3-times/week track walking & stationary bike riding	Exercise Only	Bi-weekly non-exercise-related contact	4 months	PeakVO2
Kitzman et al. (21)	3-times/week, 48-session exercise training	Exercise Only	Bi-weekly follow-up calls	4 months	SF-36 Physical Function Scale, PeakVO2, VAT, LVEF
Kitzman et al. (20)	3-times/week endurance training (warm-up, stimulation, recovery)	Exercise Only	Bi-weekly 16-week telephone interviews	4 months	6MWT, SF-36 Physical Function Scale, PeakVO2, VAT, LVEF
Kitzman et al. (22)	6-month, 36-course outpatient training (balance, strength, mobility, endurance), home exercise, personalized prescription, 4-week phone follow-up	Comprehensive CR	Bi-weekly phone follow-up, 1-month & 3-month outpatient visits	3,6 months	Deaths, 6MWT
Lang et al. (23)	Progressive exercise training (walking plan or chair exercise DVD or both)	Comprehensive CR	Routine medical management	3,6 months	Rehospitalizations
Maldonado—Martín et al. (24)	3-times/week, 60 min exercise training	Exercise Only	Attention control	4 months	6MWT, PeakVO2
Mueller et al. (25)	HIIT group: 3-times/week, 38 min sessions (12 months); MCT group: 5-times/week, 40 min sessions (12 months)	Exercise Only	12-month guideline-based physical activity recommendation	3,12 months	PeakVO2, LVAI
Palau et al. (27)	12-week, twice—daily, 20 min threshold inspiratory muscle training	Exercise Only	Weekly MIP measurement	3 months	6MWT, PeakVO2, LVEF, LVAI
Palau et al. (26)	12-week, twice-daily, 20 min threshold inspiratory muscle training (adjusted resistance)	Exercise Only	Weekly maximum inspiratory oral pressure measurement	3,6 months	PeakVO2, LVAI
Smart et al. (28)	16-week, 3-times/week, 30 min outpatient bicycle ergometer training	Exercise Only	Maintain daily activity	4 months	PeakVO2, LVEF
Sowa et al. (29)	HIIT group: 3-times/week, 38 min sessions; MCT group: 5-times/week, 40 min sessions (1–3 months supervised, 4–12 months home-based)	Exercise Only	Guideline-based exercise recommendations	3,12 months	PeakVO2

CR, Cardiac Rehabilitation; HIIT, High-Intensity Interval Training; MCT, Moderate Continuous Training.

Intervention Type: Exercise Only denotes interventions whose core component was solely structured exercise training (aerobic, resistance, interval, or inspiratory). Comprehensive CR denotes interventions that, in addition to exercise, included structured components such as patient education, counselling, risk factor management, or multidisciplinary support.

Follow-up: Duration from baseline to final outcome assessment. Values originally reported in weeks have been converted to months (approximated as 4 weeks/month).

### 3.3 Quality and bias assessment of included studies

**Figure 2:**
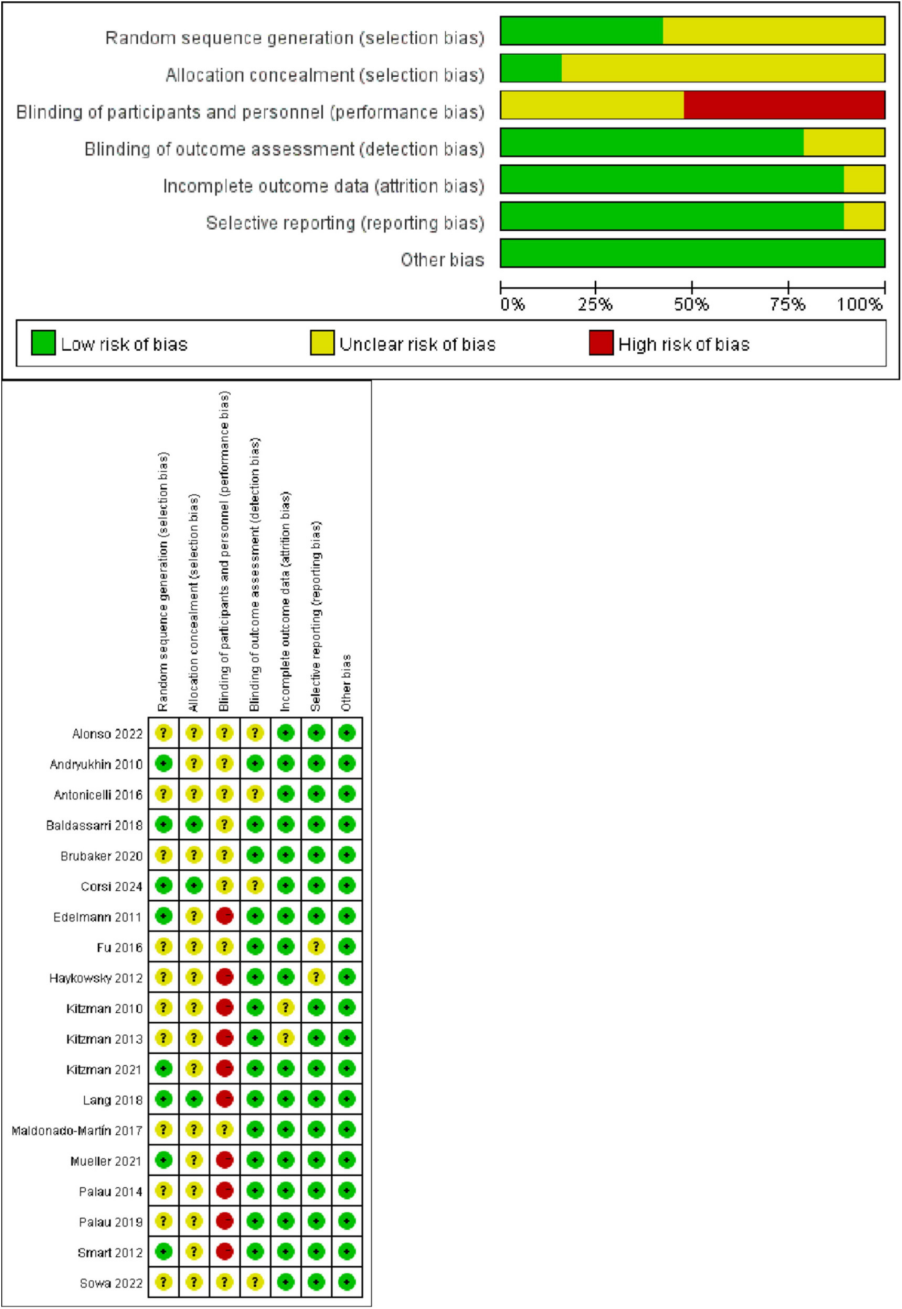
Risk assessment of bias and graphical representation of methodological quality assessment.

The methodological quality of the included trials was assessed using the Cochrane Risk of Bias tool, with the results illustrated in Figure 2. All included studies employed a randomized design. Among them, fourteen trials provided explicit descriptions of random sequence generation, thereby reducing the risk of selection bias. Allocation concealment was clearly reported in seven studies. Due to the nature of exercise-based interventions, blinding of participants and intervention personnel was often not feasible, resulting in a relatively high risk of performance bias in this domain. In contrast, the risk of detection bias associated with blinding of outcome assessors was generally low across studies. Outcome data completeness was unclear in three trials, while the remaining studies demonstrated adequate handling of missing data. With respect to other potential sources of bias, the majority of included trials were judged to be at low risk overall.

### 3.4 Baseline medication use

Baseline medication use was incompletely reported, with fewer than nineteen included randomized controlled trials providing detailed information on pharmacological therapy (Table 3). Among the studies that reported medication data, commonly used agents included beta-blockers, with usage rates ranging from 43.9% to 92%, angiotensin-converting enzyme inhibitors or angiotensin receptor blockers(25%–100%), diuretics(34.4%–100%), statins(27.9%–72.9%), and calcium channel blockers (5.3%–34.9%). Three trials—specifically those by Antonicelli et al. (13), Kitzman et al. (22), and Maldonado-Martín et al. (24)—referred only to “standard heart failure therapy” without providing detailed medication breakdowns.

**Table 3:** Baseline medication Use.

Study	Total number of subjects in group (I/C)	Beta-Blockers (I/C)	ACEI/ARB (I/C)	Diuretics (I/C)	Statins (I/C)	Calcium antagonists (I/C)
Alonso (10)	25/34	92%/94.1%	80%/82.4%	NR	NR	NR
Andryukhin (11)	44/41	61%/66%	100%/100%	100%/100%	27%/30%	27%/30%
Antonicelli R (12)	150/163	NR	NR	NR	NR	NR
Baldassarri (13)	43/19	46.5%/63.2%	69.8%/57.9%	55.8%/42.1%	27.9%/26.3%	34.9%/5.3%*
Brubaker (14)	48/51	87.5%/88.2%	79.2%/78.4%	60.4%/60.8%	72.9%/72.5%	NR
Corsi (15)	149/155	91.3%/90.0%	75.2%/74.8%	85.9%/85.2%	NR	28.2%/24.6%
Edelmann (16)	41/19	43.9%/42.1%	39.0%/36.8%	51.2%/52.6%	NR	NR
Fu (17)	30/30	73.3%/76.7%	63.3%/66.7%	50.0%/53.3%	56.7%/60.0%	NR
Haykowsky (18)	22/18	68.2%/66.7%	59.1%/55.6%	40.9%/44.4%	NR	NR
Kitzman (20)	24/22	58.3%/54.5%	50.0%/45.5%	58.3%/59.1%	41.7%/40.9%	NR
Kitzman (19)	32/31	9.4%/35.5%*	25.0%/29.0%	34.4%/32.3%	NR	NR
Kitzman (21)	175/174	NR	NR	NR	NR	NR
Lang (22)	22/23	72.7%/73.9%	63.6%/65.2%	NR	NR	NR
Maldonado-Martín et al. (23)	23/24	NR	NR	NR	NR	NR
Mueller (24)	58/60	70.7%/71.7%	63.8%/65.0%	48.3%/48.3%	NR	NR
Palau (26)	14/12	57.1%/58.3%	50.0%/50.0%	42.9%/41.7%	NR	NR
Palau (25)	13/13	69.2%/61.5%	61.5%/53.8%	53.8%/46.2%	NR	NR
Smart (27)	12/13	58.3%/46.2%	50.0%/38.5%	NR	NR	NR
Sowa (28)	24/10	70.8%/60.0%	62.5%/50.0%	50.0%/40.0%	58.3%/50.0%	NR

I/C, Intervention group/Control group; NR, Not reported;.

* Between-group difference with *P* < 0.05;.

Overall, baseline medication use appeared well balanced between intervention and control groups in most studies. One exception was reported by Baldassarri et al. (14), who observed a significant between-group difference in calcium channel blocker use. Subgroup analyses demonstrated that variations in baseline pharmacological therapy did not significantly modify the effects of exercise-based interventions on the analyzed outcomes, as indicated by non-significant subgroup interaction tests (all *P* values > 0.05; see Supplementary File S1).

### 3.5 Meta-analysis results

#### 3.5.1 Mortality and rehospitalization rates

**Figure 3:**
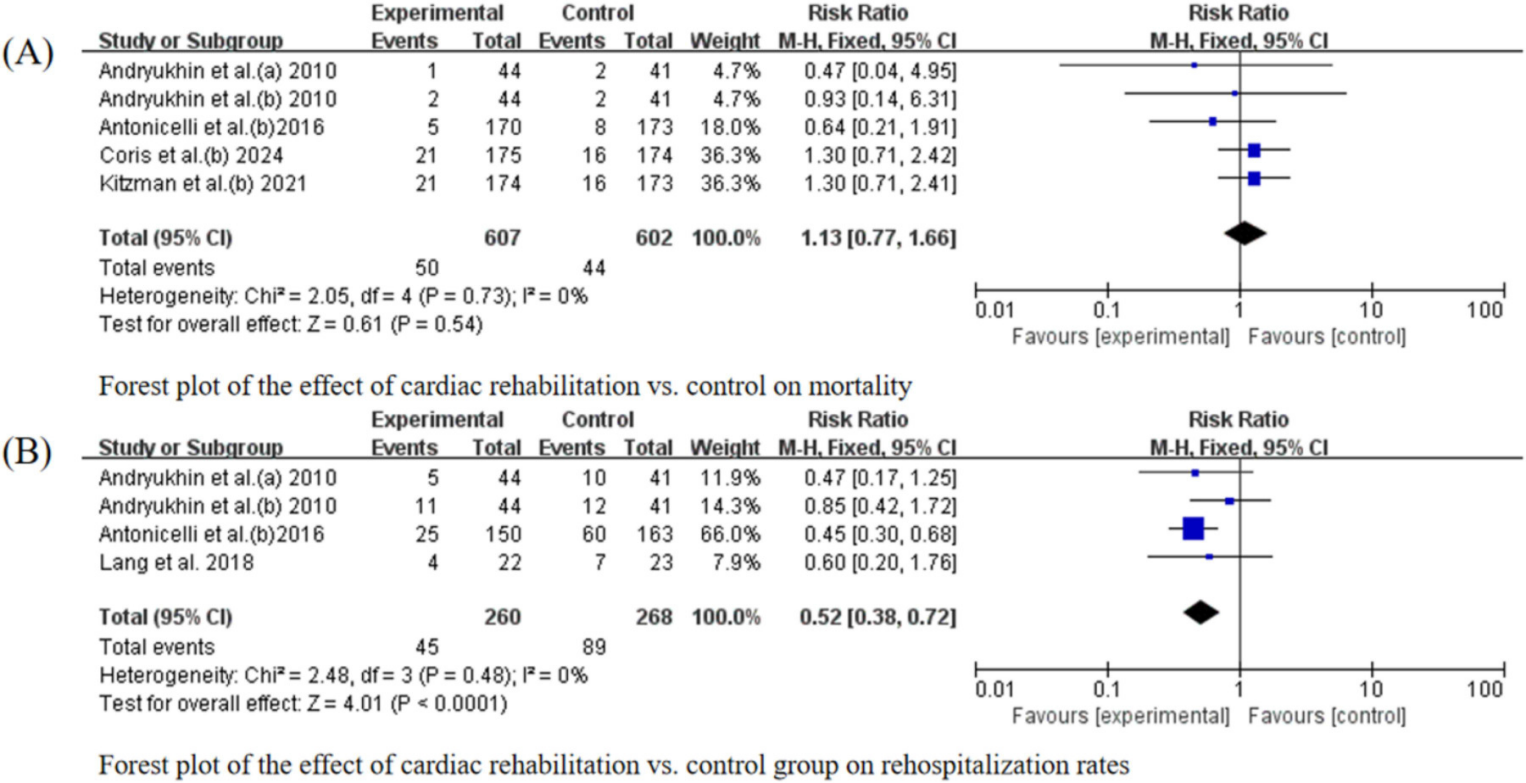
Forest plot: cardiac rehabilitation vs. Control. **(A)** Mortality; **(B)** Rehospitalization rates.

The analysis outcomes regarding mortality (Figure 3A) indicated that (12, 13, 16, 22), compared to the control group, exercise training-based cardiac rehabilitation did not demonstrate a statistically significant difference in mortality rates among HFpEF patients (overall risk ratios of 1.13, 95% CI [0.77, 1.66]; heterogeneity test *P* = 0.73, I^2^ = 0% using a fixed-effect model). Conversely (12, 13, 23), cardiac rehabilitation significantly reduced the risk of rehospitalization (Figure 3B). The pooled RR for rehospitalization was 0.52 (95%CI:0.38–0.72), with a fixed-effect model showing low heterogeneity (I^2^ = 0%). Exercise training-based interventions were associated with a reduced risk of hospitalization compared to standard care.

#### 3.5.2 6MWT

**Figure 4:**
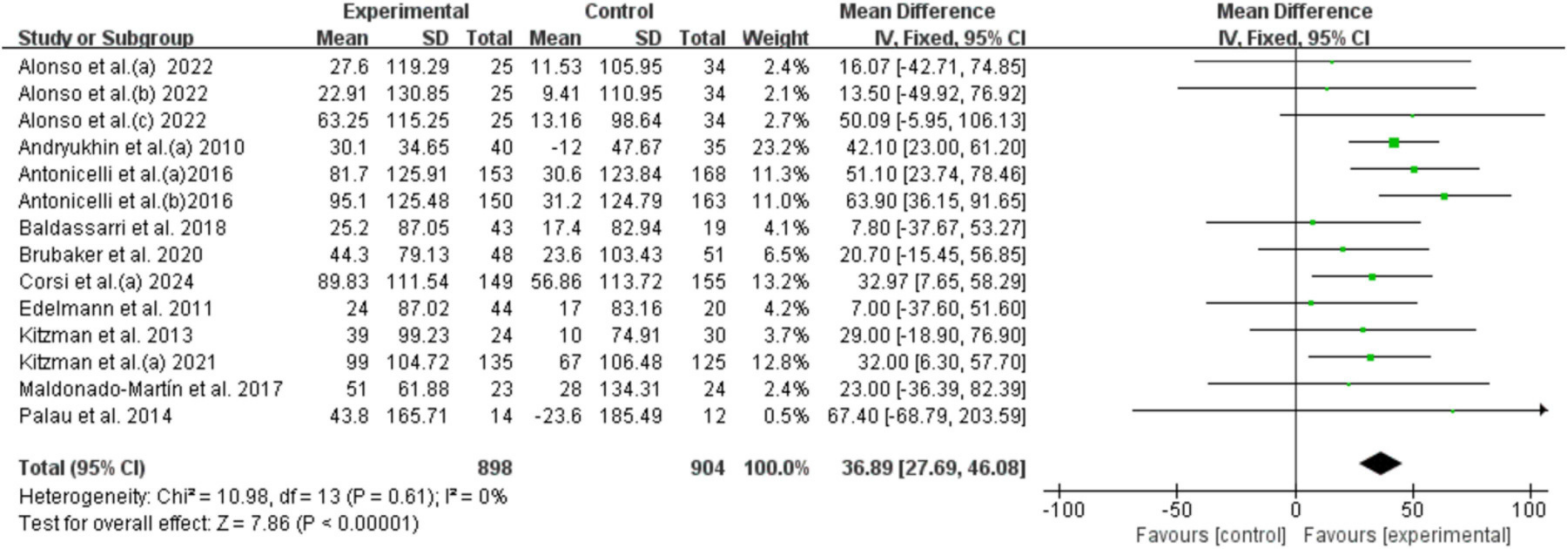
Forest plot: cardiac rehabilitation vs. Control on 6MWT*; 6MWT**:6 Min Walk Distance.

Exercise-based rehabilitation showed a significant improvement in functional exercise capacity, as measured by the 6MWD (11–17, 20, 22, 24, 27). The MD was 36.89 meters (95%CI: 27.69–46.08; *Z* = 7.86, *P* < 0.00001) (Figure 4), with negligible heterogeneity (I^2^ = 0%). This result indicates that exercise training substantially improved walking capacity compared to standard care.

**Figure 5:**
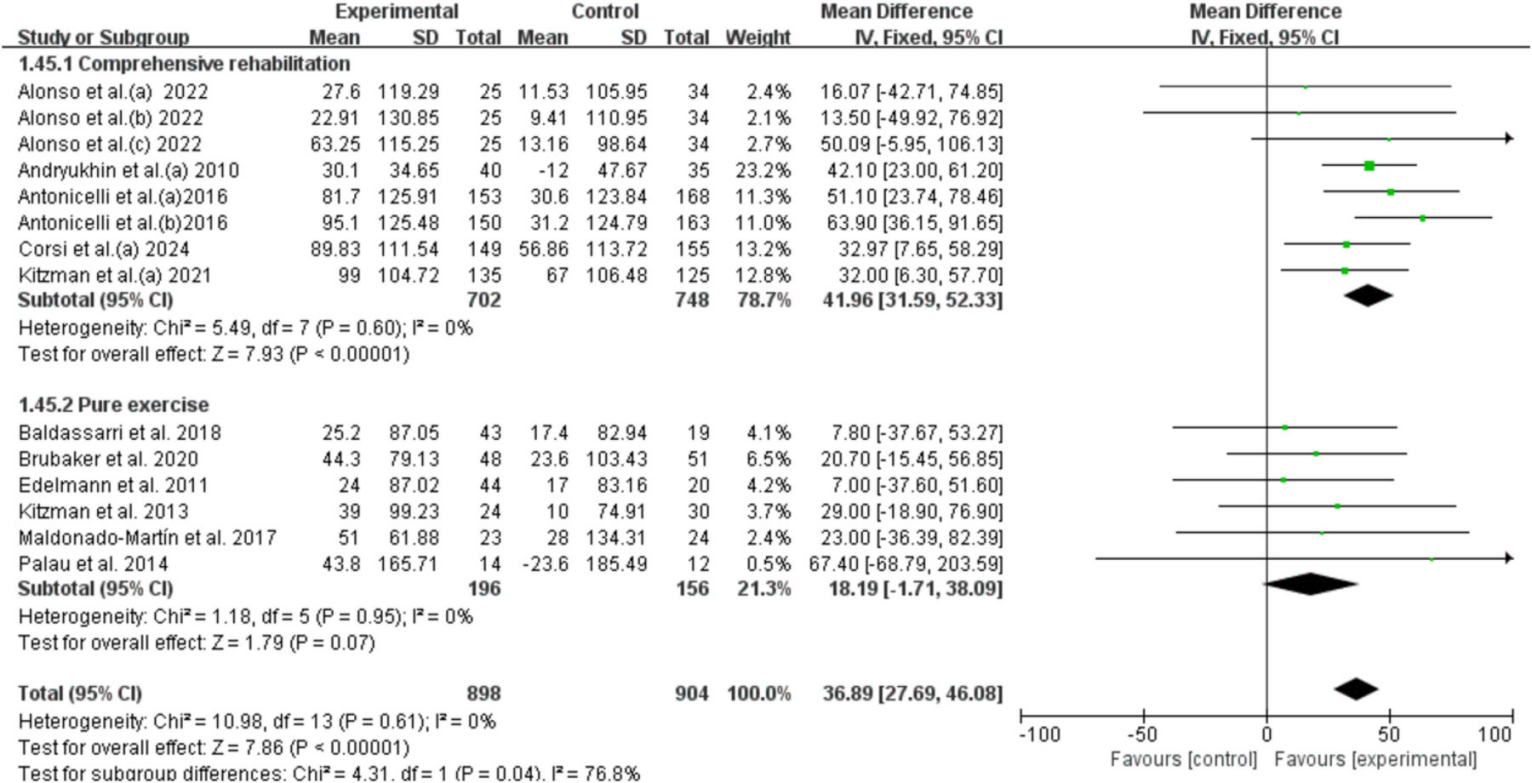
Subgroup analysis of 6MWT between control and cardiac rehabilitation groups, stratified by follow-up duration.

**Figure 6:**
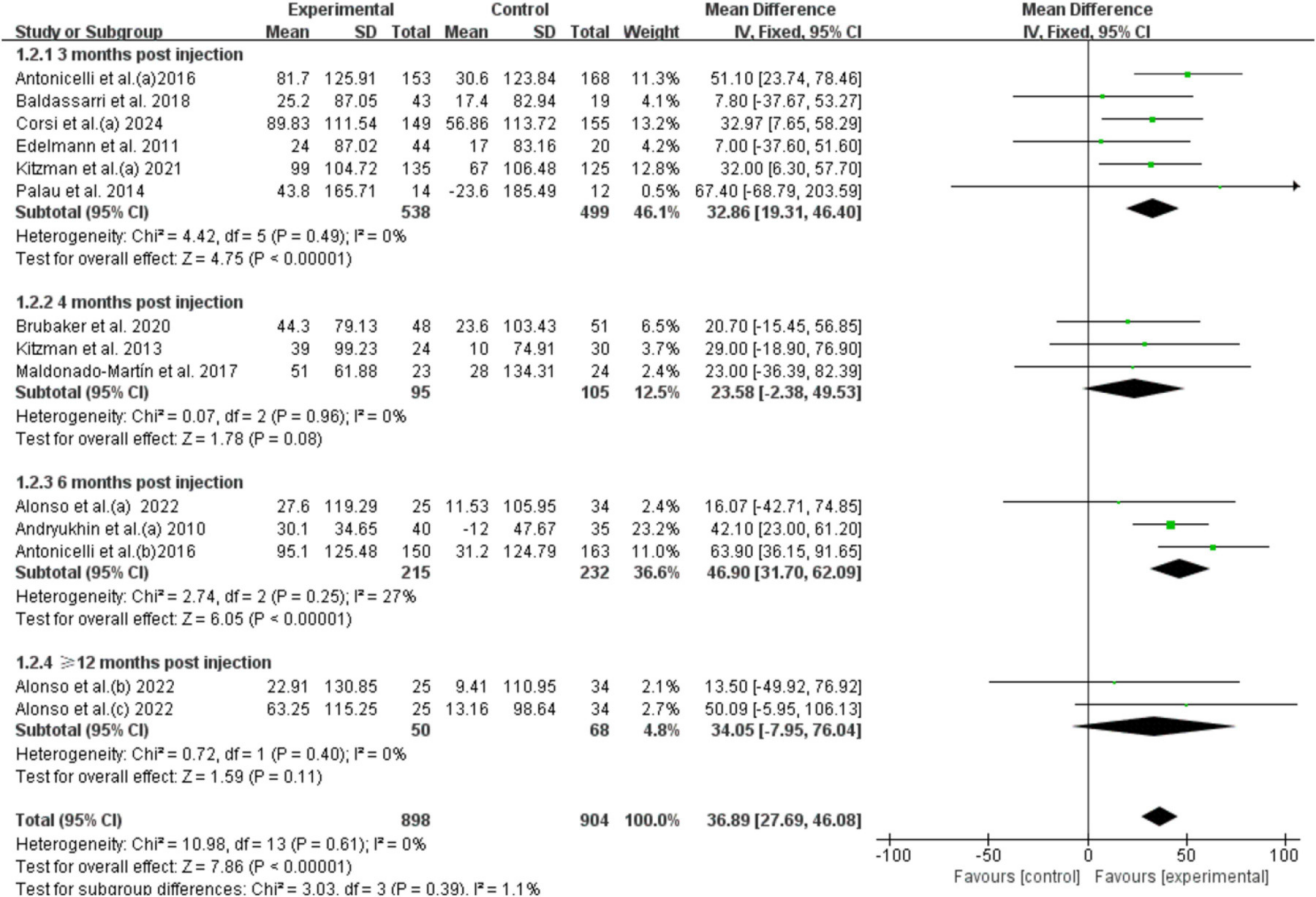
Subgroup analysis of the control group and cardiac rehabilitation group for 6MWT based on intervention complexity.

Pre-specified subgroup analyses were conducted to explore potential effect modifiers. First, stratification by intervention complexity revealed a statistically significant improvement in 6MWD for comprehensive CR programs (integrating education and multidisciplinary support), with an MD of 41.96 m (95%CI:31.59 to 52.33; *P* < 0.00001) (Figure 5). In contrast, exercise-only interventions showed a positive trend but did not achieve statistical significance (MD = 18.19 m, 95%CI:−1.71 to 38.09; *P* = 0.07). The test for subgroup differences indicated that the effect of comprehensive CR was statistically greater than that of exercise-only interventions (Chi^2^ = 4.31, df = 1, *P* = 0.04; I^2^ = 76.8%). Second, subgroup analysis based on follow-up duration showed that the significant benefit was sustained at 3 months (MD = 32.86 m, 95% CI:19.31 to 46.40; *P* < 0.00001) and 6 months (MD = 46.90 m, 95% CI: 31.70 to 62.09; *P* < 0.00001) (Figure 6). The point estimates at 4 months (MD = 23.58 m, 95% CI: −2.38 to 49.53) and ≥12 months (MD = 34.05 m, 95%CI: −7.95 to 76.04) also favored CR but did not reach statistical significance, likely due to wider confidence intervals from smaller sample sizes in these subgroups. The test for differences across follow-up times was not significant (Chi^2^ = 3.03, df = 3, *P* = 0.39; I^2^ = 1.1%).

#### 3.5.3 SF-36 physical functioning scale

**Figure 7:**
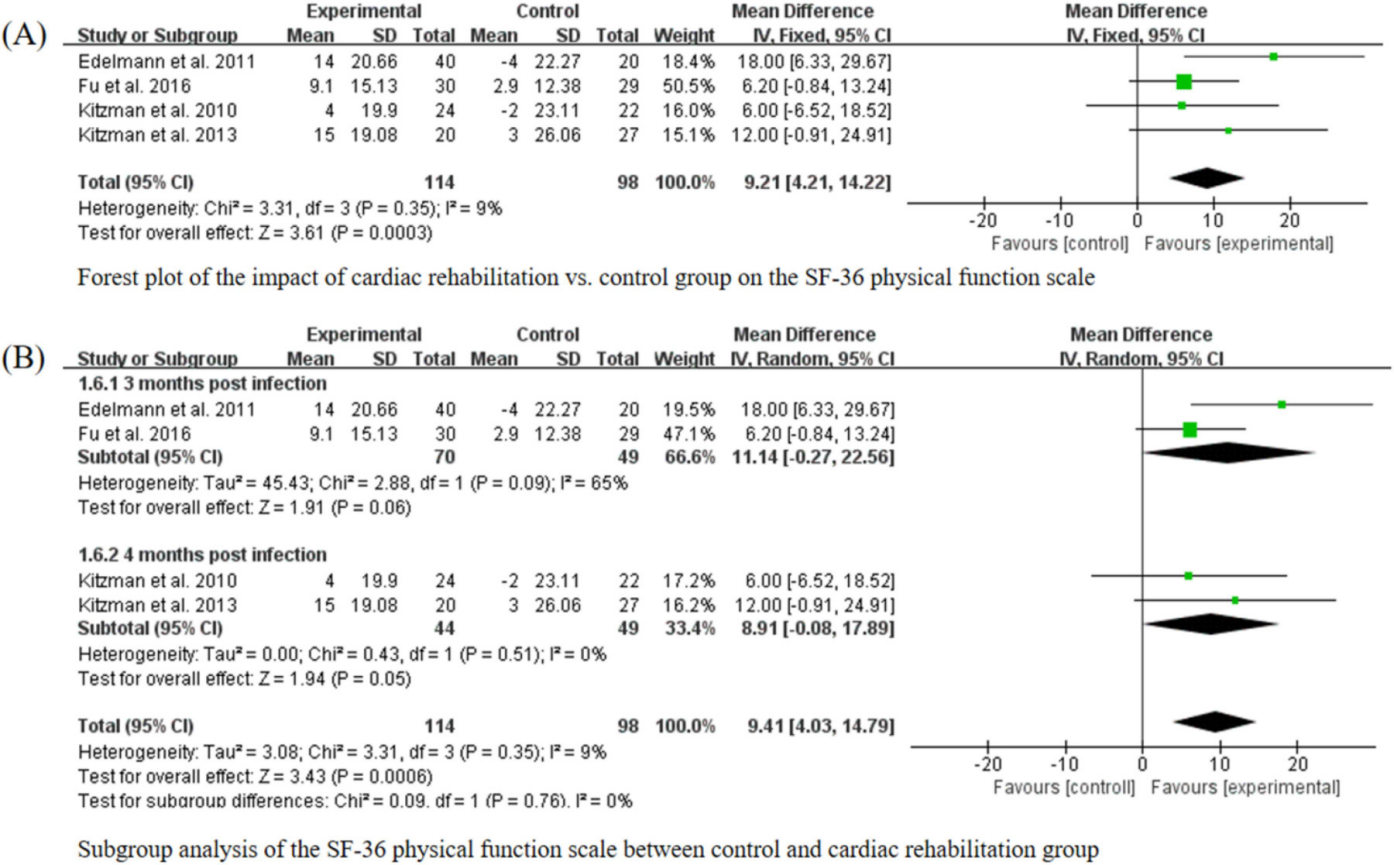
Forest plot: cardiac rehabilitation vs. Control. **(A)** SF-36 physical function scale; **(B)** Subgroup analysis by follow-up time.

Four studies with 242 participants (144 in the intervention group and 98 in the control group) reported on the SF-36 Physical Functioning Scale (17, 18, 20, 21) (Figure 7A)The pooled analysis demonstrated that exercise-based cardiac rehabilitation significantly improved physical functioning and overall quality of life compared to usual care, with a MD of 9.21 points (95% CI: 4.21–14.22; *Z* = 3.61, *P* = 0.0003). The heterogeneity among the studies was low (I^2^ = 9%, *P* = 0.35), and a fixed-effect model was used.

Subgroup analysis based on the timing of assessments further explored the temporal pattern of this improvement (Figure 7B). Although both the 3-month (MD = 11.14 points) and 4-month (MD = 8.91 points) post-intervention assessments favored the rehabilitation group, neither reached conventional statistical significance (*P* = 0.06 and *P* = 0.05, respectively). This lack of significance is likely due to smaller subgroup sizes, which reduced statistical power. Importantly, the test for subgroup differences indicated no significant variation between the 3-month and 4-month time points (Chi^2^ = 0.09, df = 1, *P* = 0.76; I^2^ = 0%).

#### 3.5.4 VO_2_peak and ventilatory anaerobic threshold (VAT)

**Figure 8:**
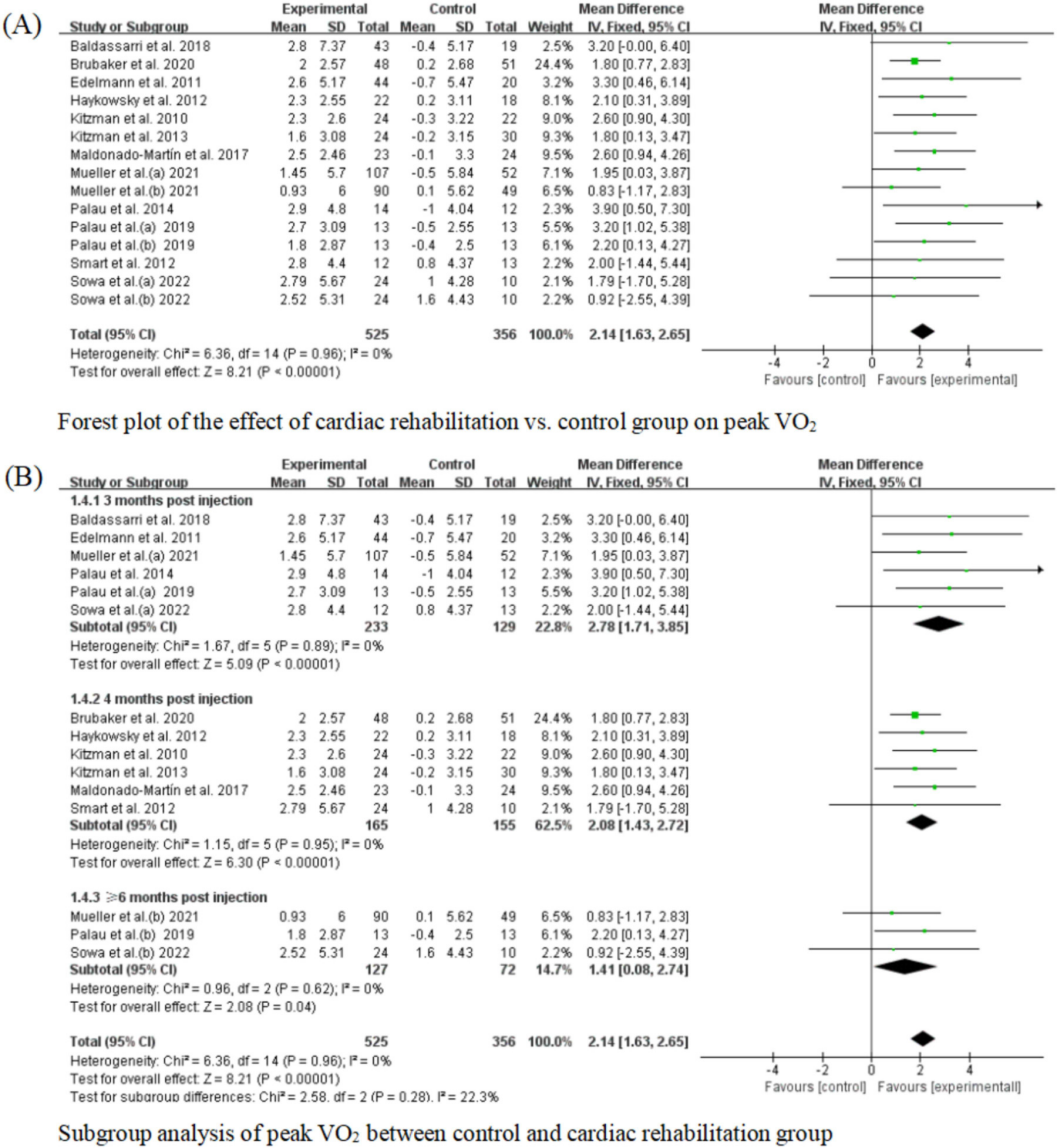
Forest plot: cardiac rehabilitation vs. Control. **(A)** Peak Oxygen Uptake; **(B)** Subgroup analysis by follow-up time. VO_2_peak, Peak Oxygen Uptake.

Fifteen studies (14, 15, 17, 19–21, 24–29), with 525 participants in the intervention group and 356 in the control group, reported data on VO₂peak (Figure 8A). The pooled analysis revealed that exercise-based cardiac rehabilitation significantly improved VO₂peak compared to the control group, with a mean difference (MD) of 2.14 mL/kg/min (95% CI:1.63 to 2.65; *Z* = 8.21, *P* < 0.00001). There was negligible heterogeneity among the studies (I^2^ = 0%, *P* = 0.96), so a fixed-effect model was used.

Subgroup analysis based on follow-up time further elucidated this benefit (Figure 8B). Significant improvements in VO₂peak were observed at both the 3-month (MD = 2.78 mL/kg/min, 95% CI: 1.71 to 3.85; *P* < 0.00001) and 4-month (MD = 2.08 mL/kg/min, 95% CI: 1.43 to 2.72;*P* < 0.00001) follow-up assessments. The effect was sustained at ≥6 months (MD = 1.41 mL/kg/min, 95% CI: 0.08 to 2.74; *P* = 0.04), although the magnitude of improvement was smaller at this time point. Statistical testing revealed no significant difference in the effect across time points (Chi^2^ = 2.58, df = 2, *P* = 0.28; I^2^ = 22.3%).

**Figure 9:**
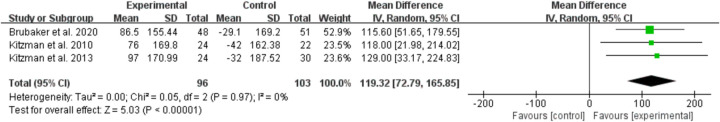
Forest plot: cardiac rehabilitation vs. Control on Ventilatory Anaerobic Threshold. VAT, Ventilatory Anaerobic Threshold.

Additionally, three studies (15, 20, 21) reporting VAT (Figure 9) found a significant increase in anaerobic threshold favoring the CR group (MD = 119.32 mL/min, 95% CI:72.79 to 165.85). There was no heterogeneity (I^2^ = 0%), and the results were highly significant (*Z* = 5.03, *P* < 0.00001).

#### 3.5.5 LVEF and left atrial volume Index (LAVI)

**Figure 10:**
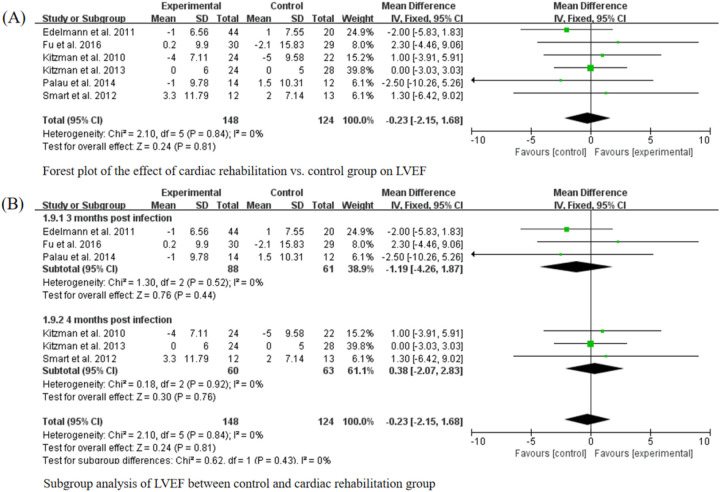
Forest plot: cardiac rehabilitation vs. Control. **(A)** Left Ventricular Ejection Fraction; **(B)** Subgroup analysis by follow-up time. LVEF, Left Ventricular Ejection Fraction.

The analysis of six studies (17, 18, 20, 21, 27, 28) (148 participants in the intervention group and 124 in the control group) that assessed LVEF (Figure 10A) found no significant effect of exercise-based cardiac rehabilitation on LVEF (MD = −0.23%, 95% CI:−2.15 to 1.68; *Z* = 0.24, *P* = 0.81). The heterogeneity was low (I^2^ = 0%). Subgroup analysis by follow-up time (Figure 10B) showed similar null results for both 3-month and 4-month assessments (*P* = 0.44 and *P* = 0.76, respectively), and the difference between subgroups was not significant (*P* = 0.43).

**Figure 11:**
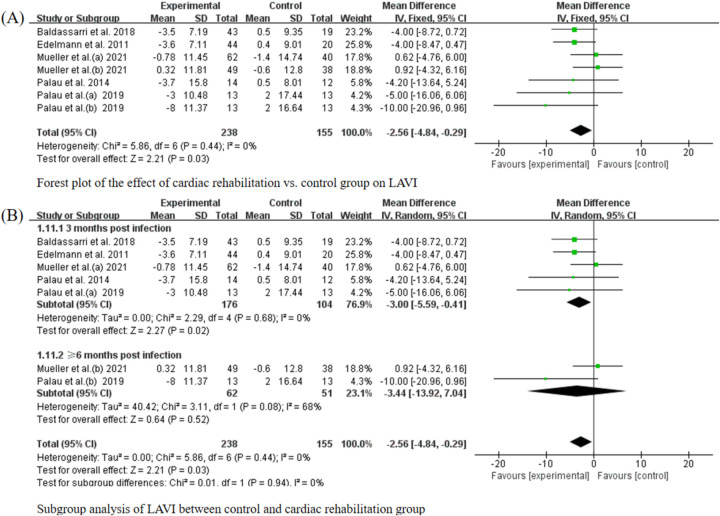
Forest plot: cardiac rehabilitation vs. Control. **(A)** Left Atrial Volume Index; **(B)** Subgroup analysis by follow-up time. LAVI, Left Atrial Volume Index.

Conversely, seven studies (14, 17, 25–27) (238 participants in the CR group, 155 controls) assessed LAVI (Figure 11A), demonstrating a significant reduction in LAVI following exercise-based rehabilitation (MD = −2.56 mL/m^2^, 95% CI: −4.84 to −0.29; *P* = 0.03). Heterogeneity was low (I^2^ = 0%). Subgroup analysis (Figure 11B) showed that the reduction in LAVI was significant at the 3-month follow-up (MD = −3.00 mL/m^2^, 95% CI: −5.59 to −0.41; *P* = 0.02), but not at ≥6 months (MD = −3.44 mL/m^2^, 95% CI: −13.92 to 7.04; *P* = 0.52). The test for subgroup differences was not significant (*P* = 0.94).

#### 3.5.6 Other diastolic function parameters and biomarkers

The E/e' ratio showed a statistically significant reduction following exercise-based rehabilitation (MD = −1.55, 95% CI:−2.58 to −0.51; *P* = 0.003), suggesting an improvement in left ventricular filling pressure. However, other parameters, such as e' velocity, E/A ratio, and E-wave and A-wave velocities, did not show significant changes(Supplementary File S2 **(Diastolic Function Parameters)**). Substantial heterogeneity was found in the analysis of e' velocity (I^2^ = 64%), but sensitivity analysis confirmed that the overall lack of significance remained unchanged.

Regarding biomarkers, data on NT-proBNP and BNP were sparse, with only two trials(Baldassarri et al. (14), Mueller et al. (25) reporting these outcomes. Due to variations in measurement protocols, a pooled analysis could not be performed. As neurohormonal activation is central to HFpEF pathophysiology (30, 31), further research is needed to explore whether exercise influences these biomarkers.

#### 3.5.7 Additional cardiometabolic outcomes

Peak heart rate during exercise testing was reported in several studies. Pooled analysis demonstrated a significant increase in peak heart rate following exercise-based cardiac rehabilitation (MD = 7.74 bpm, 95% CI: 3.68 to 11.80; *P* = 0.0002), with low heterogeneity (I^2^ = 4%) (Supplementary File S2 (**Additional Cardiometabolic Outcomes**)). Cardiometabolic outcomes such as lipid profiles and glycemic control were infrequently reported and showed substantial heterogeneity across studies; therefore, no quantitative synthesis was performed.

## 4 Discussion

This meta-analysis indicates that exercise-based CR in patients with HFpEF does not confer a statistically significant reduction in all-cause mortality, but is associated with a marked decrease in hospitalization risk, together with clinically meaningful improvements in functional exercise capacity, cardiopulmonary fitness, and physical aspects of health-related quality of life. Importantly, the pre-specified subgroup analyses offer additional insight into factors that may modify these effects, particularly with respect to intervention complexity and the temporal persistence of benefits.

The findings suggest that cardiac rehabilitation, with exercise training as its central component, plays an important role in reducing rehospitalization among patients with HFpEF. This effect is likely mediated through several interacting mechanisms. Structured exercise improves functional capacity, skeletal muscle metabolism, and cardiovascular reserve, enhancing patients' tolerance to physiological stress and reducing susceptibility to decompensation events (10). In addition, exercise-related improvements in diastolic filling characteristics and myocardial compliance may reduce left ventricular filling pressures, thereby alleviating congestion-related symptoms that commonly precipitate hospital admissions (32). Beyond physiological adaptations, exercise-based CR has demonstrated favorable effects on psychological well-being, including reductions in anxiety and depressive symptoms (33). Improved mental health may facilitate better self-care behaviors, medication adherence, and symptom monitoring, collectively contributing to lower hospitalization risk. These effects are particularly relevant in older HFpEF populations, where frailty and multimorbidity frequently drive recurrent admissions. As highlighted by Murad and Kitzman (34) exercise-based interventions may mitigate frailty-related functional decline, indirectly reducing rehospitalization risk in elderly patients with heart failure.

In contrast, this analysis did not demonstrate a significant effect of cardiac rehabilitation on mortality among patients with HFpEF. Several factors may account for this observation. First, the overall sample size across the included studies was relatively limited, and follow-up durations were generally short, restricting the ability to detect long-term survival effects. Cardiovascular disease progression and mortality risk evolve over extended periods, and short-term follow-up may not adequately capture the downstream impact of exercise interventions on survival. Second, mortality in HFpEF is driven by a complex interplay of causes, including non-cardiovascular comorbidities (35), which may dilute the observable effect of exercise training on all-cause mortality. As noted by Cheng et al. (36), patients with HFpEF often present with multiple interrelated chronic conditions, substantially complicating mortality risk assessment and making it challenging to isolate the contribution of cardiac rehabilitation. Consistent with these findings, earlier work by Taylor et al. did not demonstrate a clear mortality benefit of exercise training in HFpEF, largely due to constraints related to sample size and study design (37). Although the study by Molloy et al. did not observe a short-term reduction in all-cause mortality (38), its larger cohort and inclusion of diverse heart failure phenotypes suggest that exercise-based CR may still exert favorable effects on long-term survival, warranting further investigation.

Functional capacity and quality of life are central clinical outcomes in HFpEF, and both the 6MWT and the SF-36 physical functioning scale serve as key indicators in this regard. In line with previous reports (39–43), the present analysis confirms that cardiac rehabilitation significantly improves performance on both measures, with findings that were consistent and robust across studies. Subgroup analyses further clarified these effects. Comprehensive CR programs that combined structured exercise with patient education, lifestyle counseling, and multidisciplinary support achieved significantly greater improvements in 6 min walk distance compared with interventions focused solely on exercise. This observation suggests that non-exercise components may enhance the physiological benefits of training by fostering behavioral change, improving disease self-management, and increasing long-term adherence. Moreover, functional improvements were evident across both short- and medium-term follow-up periods, indicating that the benefits of CR extend beyond the immediate intervention phase. Supporting these findings, Guo Yuan et al. reported that exercise-based cardiac rehabilitation significantly improves quality of life in patients with HFpEF compared with conventional treatment approaches (40). Clinically, the enhancement of physical performance and functional independence is particularly meaningful in HFpEF, where symptom burden and activity limitation often outweigh survival considerations in determining patient well-being.

Exercise intolerance in HFpEF is multifactorial, arising from complex interactions among cardiac, vascular, and skeletal muscle abnormalities (44). Reduced VO₂peak and VAT are hallmark features of the condition. The present analysis demonstrates that exercise-based CR significantly improves both VO₂peak and VAT, reflecting enhanced aerobic capacity and metabolic efficiency. Mechanistically, HFpEF is associated with skeletal muscle myopathy, mitochondrial dysfunction, altered fiber composition, and impaired oxidative metabolism (45). Peripheral vascular dysfunction and increased arterial stiffness further limit oxygen delivery during exertion (46–48). As described by Sarma et al. (49, 50), patients with HFpEF exhibit impaired oxidative capacity, abnormal phosphocreatine metabolism, and increased reliance on anaerobic glycolysis, contributing to reduced exercise tolerance. Exercise training can counteract these abnormalities by promoting mitochondrial biogenesis, increasing oxidative enzyme activity, enhancing capillary density, and improving muscle strength and endurance (5). The persistence of VO₂peak improvements across different follow-up intervals observed in this study suggests that these adaptations are not merely transient. Although some studies have suggested potential improvements in endothelial function with exercise training (51, 52), the current analysis could not robustly confirm this effect due to inconsistent reporting of vascular outcomes. Nonetheless, the observed increases in VAT indicate improved efficiency of aerobic metabolism, allowing patients to perform submaximal activities with less fatigue. These findings are consistent with prior work by Pandey et al. (10), reinforcing the role of exercise-based CR in addressing the core mechanisms of exercise intolerance in HFpEF. In addition, the observed increase in peak heart rate further supports the beneficial effects of exercise training on cardiovascular functional reserve and chronotropic competence in patients with HFpEF.

LAVI is widely recognized as an indicator of chronic diastolic burden and adverse cardiac remodeling in HFpEF (53). This meta-analysis identified a modest but statistically significant reduction in LAVI following exercise-based CR, suggesting a favorable effect on left atrial remodeling and diastolic filling pressures. Chronic inflammation, endothelial dysfunction, and myocardial fibrosis are key contributors to diastolic impairment in HFpEF (53–55), and exercise training may mitigate these processes through anti-inflammatory effects and improvements in hemodynamic loading conditions. In contrast, left ventricular ejection fraction (LVEF) did not change significantly following CR, a finding consistent with previous meta-analyses (10). This result is not unexpected. HFpEF is characterized primarily by diastolic dysfunction, while systolic function is generally preserved, limiting the potential for exercise interventions to influence LVEF. Furthermore, as noted by Haykowsky et al. (56), LVEF is determined by a complex interplay of myocardial structure, diastolic properties, and loading conditions. The mechanisms through which exercise training might influence LVEF in HFpEF remain poorly defined and are likely multifactorial, involving both central cardiovascular and peripheral skeletal muscle adaptations.

Several limitations of this study should be acknowledged. First, the risk-of-bias assessment identified methodological weaknesses in some included trials, particularly with respect to randomization procedures, allocation concealment, and blinding, which may affect the internal validity of the findings. Second, although random-effects models were employed to account for heterogeneity, substantial variability persisted for certain outcomes, likely reflecting differences in patient characteristics and intervention protocols. Second, several studies were constrained by small sample sizes, limiting statistical power and the precision of pooled estimates. Variability in follow-up duration, ranging from several months to more than a year, may also have influenced the evaluation of longer-term effects. Given the limited availability of large-scale trials with extended follow-up, the durability of CR benefits in HFpEF remains uncertain, highlighting the need for future high-quality studies with larger cohorts and longer observation periods. Third, the lack of standardized reporting of cardiometabolic outcomes and absence of patient-level subgroup data limited the ability to explore mechanistic pathways and identify populations most likely to benefit.

An additional limitation relates to the incomplete reporting of baseline guideline-directed medical therapy (GDMT) across the included trials. Although contemporary HFpEF management emphasizes the prognostic importance of pharmacological therapies such as ACEIs/ARBs, beta-blockers, and SGLT2 inhibitors, medication use was often insufficiently detailed and frequently described only as “standard therapy”. This lack of granularity introduces potential confounding and may compromise the interpretability of the results. For example, unreported differences in diuretic use could influence functional outcomes attributed to exercise training. Nevertheless, the observed consistency of exercise-related benefits across diverse patient populations suggests that the positive effects of CR may be largely independent of background medical therapy. However, given the extent of missing medication data, this inference should be regarded as preliminary and requires confirmation in future studies with standardized and comprehensive reporting of pharmacological treatment.

Despite these limitations, the findings of this study have important clinical implications. Clinically, exercise-based cardiac rehabilitation should be integrated into HFpEF management. Comprehensive, multidisciplinary programs may offer greater benefits than exercise alone. Moderate-intensity continuous training remains the most commonly applied modality, while rehabilitation strategies should be individualized according to patient characteristics, comorbidities, and exercise tolerance, with supervised programs potentially benefiting older or frail patients.

## 5 Conclusion

This meta-analysis suggests that exercise-based cardiac rehabilitation improves functional capacity, cardiorespiratory fitness, and physical quality of life in patients with HFpEF, while significantly reducing rehospitalization risk. Although no mortality benefit was observed, these functional gains address key clinical priorities in HFpEF management. However, the effects on cardiometabolic outcomes remain uncertain due to limited and inconsistent reporting, highlighting the need for further high-quality studies.
