# Supplementary material for: The impact of cardiac rehabilitation on rehospitalization and mortality rates in heart failure with preserved ejection fraction
Source: Front Cardiovasc Med. 2026 May 28;13:1799035. doi: 10.3389/fcvm.2026.1799035 (PMC13253794; doi:10.3389/fcvm.2026.1799035)
Supplement: Supplementary file 1 [file Datasheet1.pdf]

Diastolic Function Parameters

1.e' velocity(Early diastolic mitral annular velocity)

Study or Subgroup	Experimental Mean	Experimental SD	Experimental Total	Control Mean	Control SD	Control Total	Weight	Mean Difference IV, Random, 95% CI
Edelmann et al. 2011	0.9	1.25	44	-0.5	1.41	20	26.5%	1.40 [0.68, 2.12]
Mueller et al.(a) 2021	-0.06	1.72	108	-0.35	1.85	53	29.9%	0.29 [-0.30, 0.88]
Mueller et al.(b) 2021	-0.11	1.71	99	-0.2	1.79	52	29.9%	0.09 [-0.50, 0.68]
Palau et al. 2014	0.5	2.06	14	0.2	1.52	12	13.7%	0.30 [-1.08, 1.68]
**Total (95% CI)**			**265**			**137**	**100.0%**	**0.53 [-0.10, 1.15]**

Heterogeneity: Tau² = 0.25; Chi² = 8.40, df = 3 (P = 0.04); I² = 64%
Test for overall effect: Z = 1.65 (P = 0.10)

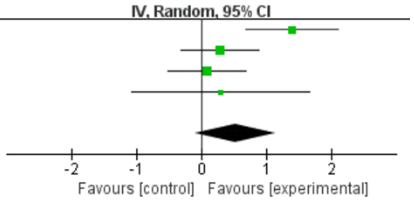

2.e' velocity(Early diastolic mitral annular velocity) sensitivity analysis

Study or Subgroup	Experimental Mean	Experimental SD	Experimental Total	Control Mean	Control SD	Control Total	Weight	Mean Difference IV, Random, 95% CI
Edelmann et al. 2011	0.9	1.25	44	-0.5	1.41	20	0.0%	1.40 [0.68, 2.12]
Mueller et al.(a) 2021	-0.06	1.72	108	-0.35	1.85	53	45.6%	0.29 [-0.30, 0.88]
Mueller et al.(b) 2021	-0.11	1.71	99	-0.2	1.79	52	46.0%	0.09 [-0.50, 0.68]
Palau et al. 2014	0.5	2.06	14	0.2	1.52	12	8.5%	0.30 [-1.08, 1.68]
**Total (95% CI)**			**221**			**117**	**100.0%**	**0.20 [-0.20, 0.60]**

Heterogeneity: Tau² = 0.00; Chi² = 0.24, df = 2 (P = 0.89); I² = 0%
Test for overall effect: Z = 0.97 (P = 0.33)

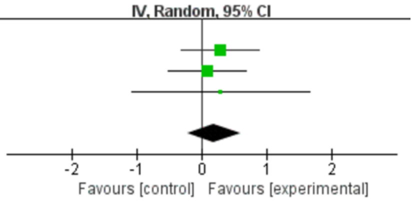

3.E/e' ratio(Ratio of early transmitral flow velocity to early diastolic mitral annular velocity)

Study or Subgroup	Experimental Mean	Experimental SD	Experimental Total	Control Mean	Control SD	Control Total	Weight	Mean Difference IV, Fixed, 95% CI
Edelmann et al. 2011	-2.3	2.91	44	0.6	4.29	20	25.1%	-2.90 [-4.97, -0.83]
Fu et al. 2016	-4.9	11.12	30	-1.4	9.46	29	3.9%	-3.50 [-8.76, 1.76]
Mueller et al.(a) 2021	-0.45	4.53	108	0.8	6.66	53	27.2%	-1.25 [-3.24, 0.74]
Mueller et al.(b) 2021	-0.91	4.2	99	0	5.67	52	35.0%	-0.91 [-2.66, 0.84]
Palau et al. 2014	-3.3	7.37	14	-5.5	12.05	12	1.7%	2.20 [-5.63, 10.03]
Palau et al.(a) 2019	-1.2	9.49	13	-0.2	3.91	13	3.4%	-1.00 [-6.58, 4.58]
Palau et al.(b) 2019	-0.8	9.44	13	1.3	5.66	13	3.0%	-2.10 [-8.08, 3.88]
Smart et al. 2012	4.4	20.8	12	0	6.25	13	0.7%	4.40 [-7.85, 16.65]
**Total (95% CI)**			**333**			**205**	**100.0%**	**-1.55 [-2.58, -0.51]**

Heterogeneity: Chi² = 4.62, df = 7 (P = 0.71); I² = 0%
Test for overall effect: Z = 2.93 (P = 0.003)

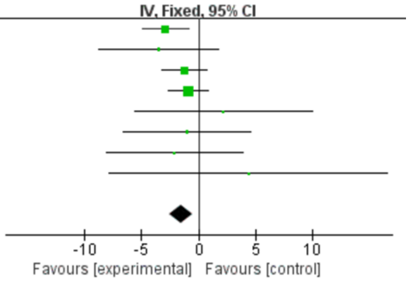

4.E/A ratio(Ratio of early to late transmitral flow velocities)

Study or Subgroup	Experimental Mean	Experimental SD	Experimental Total	Control Mean	Control SD	Control Total	Weight	Mean Difference IV, Fixed, 95% CI
Fu et al. 2016	-0.2	0.95	30	-0.2	1.46	29	3.1%	0.00 [-0.63, 0.63]
Kitzman et al. 2010	0.12	0.27	24	0.1	0.39	22	32.1%	0.02 [-0.18, 0.22]
Mueller et al.(a) 2021	-0.05	0.63	93	0.2	0.87	49	16.2%	-0.25 [-0.53, 0.03]
Mueller et al.(b) 2021	-0.06	0.69	84	0.03	0.7	44	19.0%	-0.09 [-0.34, 0.16]
Smart et al. 2012	-0.05	0.15	12	-0.12	0.34	13	29.6%	0.07 [-0.13, 0.27]
**Total (95% CI)**			**243**			**157**	**100.0%**	**-0.03 [-0.14, 0.08]**

Heterogeneity: Chi² = 3.86, df = 4 (P = 0.43); I² = 0%
Test for overall effect: Z = 0.54 (P = 0.59)

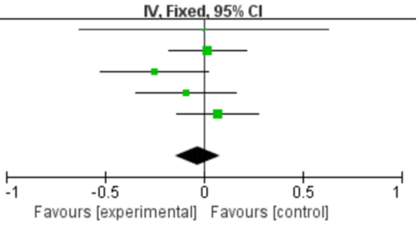

5.E-wave velocity(Early diastolic transmitral flow velocity)

Study or Subgroup	Experimental Mean	Experimental SD	Experimental Total	Control Mean	Control SD	Control Total	Weight	Mean Difference IV, Fixed, 95% CI
Kitzman et al. 2010	3	19.37	24	3	23.11	22	19.9%	0.00 [-12.38, 12.38]
Mueller et al.(a) 2021	-3.75	23.94	109	-2.1	28.48	53	38.7%	-1.65 [-10.54, 7.24]
Mueller et al.(b) 2021	-6.36	23.29	100	-2.1	26.76	52	41.4%	-4.26 [-12.85, 4.33]
**Total (95% CI)**			**233**			**127**	**100.0%**	**-2.40 [-7.93, 3.12]**

Heterogeneity: Chi² = 0.35, df = 2 (P = 0.84); I² = 0%
Test for overall effect: Z = 0.85 (P = 0.39)

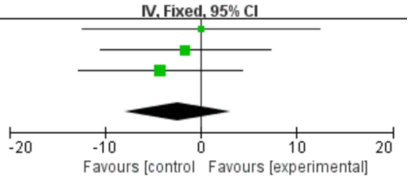

6.A-wave velocity(Late diastolic transmitral flow velocity (atrial contraction))

Study or Subgroup	Experimental Mean	Experimental SD	Experimental Total	Control Mean	Control SD	Control Total	Weight	Mean Difference IV, Fixed, 95% CI
Kitzman et al. 2010	-7	29.56	24	-3	21.22	22	17.1%	-4.00 [-18.78, 10.78]
Mueller et al.(a) 2021	-1.42	26.72	93	-7.9	26.46	49	44.3%	6.48 [-2.71, 15.67]
Mueller et al.(b) 2021	-0.954	27.93	84	-1.5	26.43	44	38.6%	0.55 [-9.29, 10.38]
**Total (95% CI)**			**201**			**115**	**100.0%**	**2.40 [-3.72, 8.51]**

Heterogeneity: Chi² = 1.61, df = 2 (P = 0.45); I² = 0%
Test for overall effect: Z = 0.77 (P = 0.44)

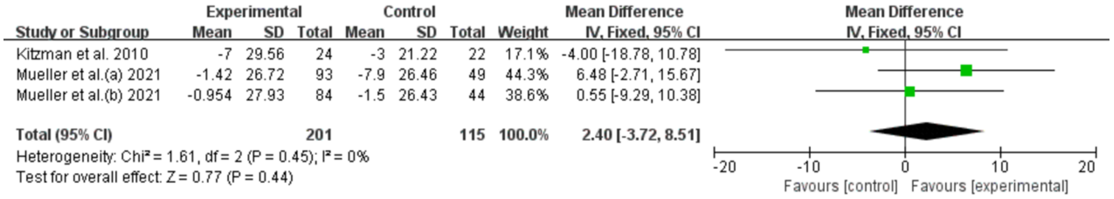

Additional Cardiometabolic Outcomes

Study or Subgroup	Experimental Mean	Experimental SD	Experimental Total	Control Mean	Control SD	Control Total	Weight	Mean Difference IV, Fixed, 95% CI
Haykowsky et al. 2012	6	19	22	-2	21.07	18	10.4%	8.00 [-4.56, 20.56]
Kitzman et al. 2010	4	18.67	26	-8	16.41	27	18.3%	12.00 [2.52, 21.48]
Kitzman et al. 2013	4	17.09	24	-4	19.31	30	17.4%	8.00 [-1.72, 17.72]
Maldonado-Martín et al. 2017	5	18.33	23	-7	18.68	24	14.7%	12.00 [1.42, 22.58]
Mueller et al.(a) 2021	4	25.87	107	0	29.48	52	18.7%	4.00 [-5.39, 13.39]
Mueller et al.(b) 2021	4	28.3	90	4	28.32	49	17.0%	0.00 [-9.85, 9.85]
Smart et al. 2012	12	22.91	12	-11	32.19	13	3.5%	23.00 [1.22, 44.78]
**Total (95% CI)**			**304**			**213**	**100.0%**	**7.74 [3.68, 11.80]**

Heterogeneity: Chi² = 6.27, df = 6 (P = 0.39); I² = 4%
Test for overall effect: Z = 3.74 (P = 0.0002)

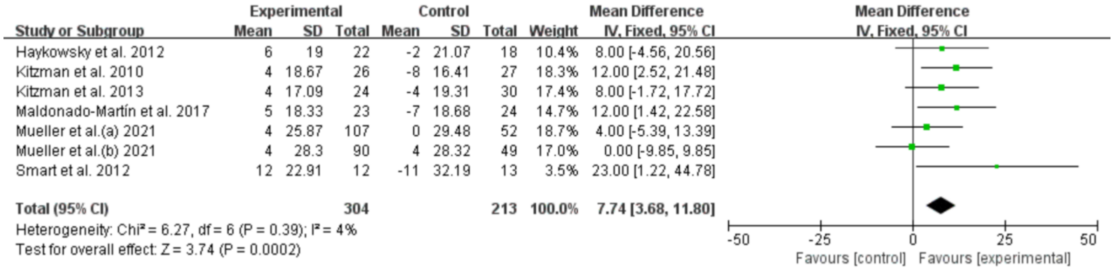

Forest plot showing the effect of exercise-based cardiac rehabilitation on peak heart rate
